# A Novel N4-Like Bacteriophage Isolated from a Wastewater Source in South India with Activity against Several Multidrug-Resistant Clinical Pseudomonas aeruginosa Isolates

**DOI:** 10.1128/mSphere.01215-20

**Published:** 2021-01-13

**Authors:** Nitasha D. Menon, Megha S. Kumar, T. G. Satheesh Babu, Sucharita Bose, Gayathri Vijayakumar, Manasi Baswe, Meghna Chatterjee, Jovita Rowena D’Silva, Kavya Shetty, Jayalekshmi Haripriyan, Anil Kumar, Samitha Nair, Priyanka Somanath, Bipin G. Nair, Victor Nizet, Geetha B. Kumar

**Affiliations:** a School of Biotechnology, Amrita Vishwa Vidyapeetham, Amritapuri, Kerala, India; b Tata Institute for Genetics and Society-India (TIGS), TIGS Center at inStem, Bangalore, Karnataka, India; c Amrita Biosensor Research Lab, Amrita Vishwa Vidyapeetham, Coimbatore, Tamil Nadu, India; d Department of Sciences, Amrita School of Engineering, Amrita Vishwa Vidyapeetham, Coimbatore, Tamil Nadu, India; e Icon Analytical Private Limited, Worli, Mumbai, Maharashtra, India; f Institute for Stem Cell Science and Regenerative Medicine, Bangalore, Karnataka, India; g Department of Microbiology, Amrita Institute of Medical Sciences, Amrita Vishwa Vidyapeetham, Kochi, Kerala, India; h Department of Microbiology, DDRC SRL Diagnostic Private Limited, Trivandrum, Kerala, India; i Department of Pharmacology, University of California, San Diego, La Jolla, California, USA; j Skaggs School of Pharmacy and Pharmaceutical Sciences, University of California, San Diego, La Jolla, California, USA; k Department of Pediatrics, University of California, San Diego, La Jolla, California, USA; University of Rochester

**Keywords:** *Pseudomonas aeruginosa*, bacteriophages, N4-like viruses, antibiotic resistance, phage therapy, clinical isolates, community-acquired infection, bacteriophage therapy

## Abstract

Multidrug-resistant community-acquired infections caused by the opportunistic human pathogen Pseudomonas aeruginosa are increasingly reported in India and other locations globally. Since this organism is ubiquitous in the environment, samples such as sewage and wastewater are rich reservoirs of P. aeruginosa bacteriophages. In this study, we report the isolation and characterization of a novel P. aeruginosa N4-like lytic bacteriophage, vB_Pae_AM.P2 (AM.P2), from wastewater in Kerala, India. AM.P2 is a double-stranded DNA podovirus that efficiently lyses the model strain, PAO1, at a multiplicity of infection as low as 0.1 phage per bacterium and resistance frequency of 6.59 × 10^−4^. Synergy in bactericidal activity was observed between AM.P2 and subinhibitory concentrations of the antibiotic ciprofloxacin. Genome sequencing of AM.P2 revealed features similar to those of the N4-like P. aeruginosa phages LUZ7 and KPP21. As judged by two independent assay methods, spot tests and growth inhibition, AM.P2 successfully inhibited the growth of almost 30% of strains from a contemporary collection of multidrug-resistant P. aeruginosa clinical isolates from South India. Thus, AM.P2 may represent an intriguing candidate for inclusion in bacteriophage cocktails developed for various applications, including water decontamination and clinical bacteriophage therapy.

**IMPORTANCE** In India, multidrug resistance determinants are much more abundant in community-associated bacterial pathogens due to the improper treatment of domestic and industrial effluents. In particular, a high bacterial load of the opportunistic pathogen P. aeruginosa in sewage and water bodies in India is well documented. The isolation and characterization of bacteriophages that could target emerging P. aeruginosa strains, representing possible epicenters for community-acquired infections, could serve as a useful alternative tool for various applications, such as phage therapy and environmental treatment. Continuing to supplement the repertoire of broad-spectrum bacteriophages is an essential tool in confronting this problem.

## Abstract (precis)

In India, multidrug resistance determinants are much more abundant in community-associated bacterial pathogens due to the improper treatment of domestic and industrial effluents. In particular, a high bacterial load of the opportunistic pathogen P. aeruginosa in sewage and water bodies in India is well documented.

## INTRODUCTION

Pseudomonas aeruginosa, an important Gram-negative opportunistic human pathogen, causes a wide range of infections, including ventilator-associated pneumonia, burn and wound infections, septicemia, and chronic pulmonary infection in cystic fibrosis patients (1). Intrinsically resistant to many antibiotics, including several β-lactam agents, P. aeruginosa is included among the ESKAPE pathogens capable of rapidly developing multidrug resistance (MDR) (2–4). While hospital-acquired infections are common throughout the world, community-acquired P. aeruginosa diseases are more likely to occur in developing countries, like India, where large human populations coexist with ineffective effluent management and a lack of adequate drinking water, providing extensive exposure and the potential for rapid dissemination.

India has an extremely high incidence of multidrug-resistant bacterial pathogens, partly attributed to the improper treatment of effluents from residential areas, pharmaceutical industries, and hospitals (5–7). Consequently, sewage and water bodies have become reservoirs for highly drug-resistant pathogens. For example, MDR P. aeruginosa is more prevalent in Indian aquatic sediments that receive hospital effluents than those in other countries (8), and carbapenem-resistant Escherichia coli and P. aeruginosa are highly abundant in sewage treatment plants (9). Rivers frequented by people from across India and the world are considered hot spots for gene transfer events culminating in antibiotic resistance (10).

An increased presence of MDR bacterial populations in the environment can lead to higher incidences of more dangerous community-acquired infections. Asia has the second highest incidence of P. aeruginosa community-acquired pneumonia in the world (11), and India has the largest proportion of community-acquired pneumonias caused by Gram-negative bacilli in Asia (12). More than 50% of P. aeruginosa clinical isolates in India are resistant to broad-spectrum fluoroquinolones and third-generation cephalosporins, with an alarming 41.8 to 46.8% of strains demonstrating carbapenem resistance (6).

Since MDR P. aeruginosa strains are prevalent in sewage and rivers in India, we concluded that bacteriophages targeting these bacteria would thrive there as well. The notion that the Ganga River could harbor antiseptic properties against certain bacteria dates back to 1896, when Hankin discovered that Ganga River water suppressed Vibrio cholerae (13). Since then, lytic bacteriophages targeting E. coli have been isolated from the Ganga River and multiple other water sources in India, including sewage (14). Here, we describe the isolation and biological characterization of the novel P. aeruginosa phage vB_Pae_AM.P2 from a wastewater source in South India. We provide the whole-genome sequence of phage AM.P2 and demonstrate its efficacy against MDR P. aeruginosa clinical isolates in consideration of its inclusion in the repertoire of potential antipseudomonal phage therapeutics.

## RESULTS

### Isolated phage AM.P2 is a P. aeruginosa-specific double-stranded DNA podovirus.

**FIG 1:**
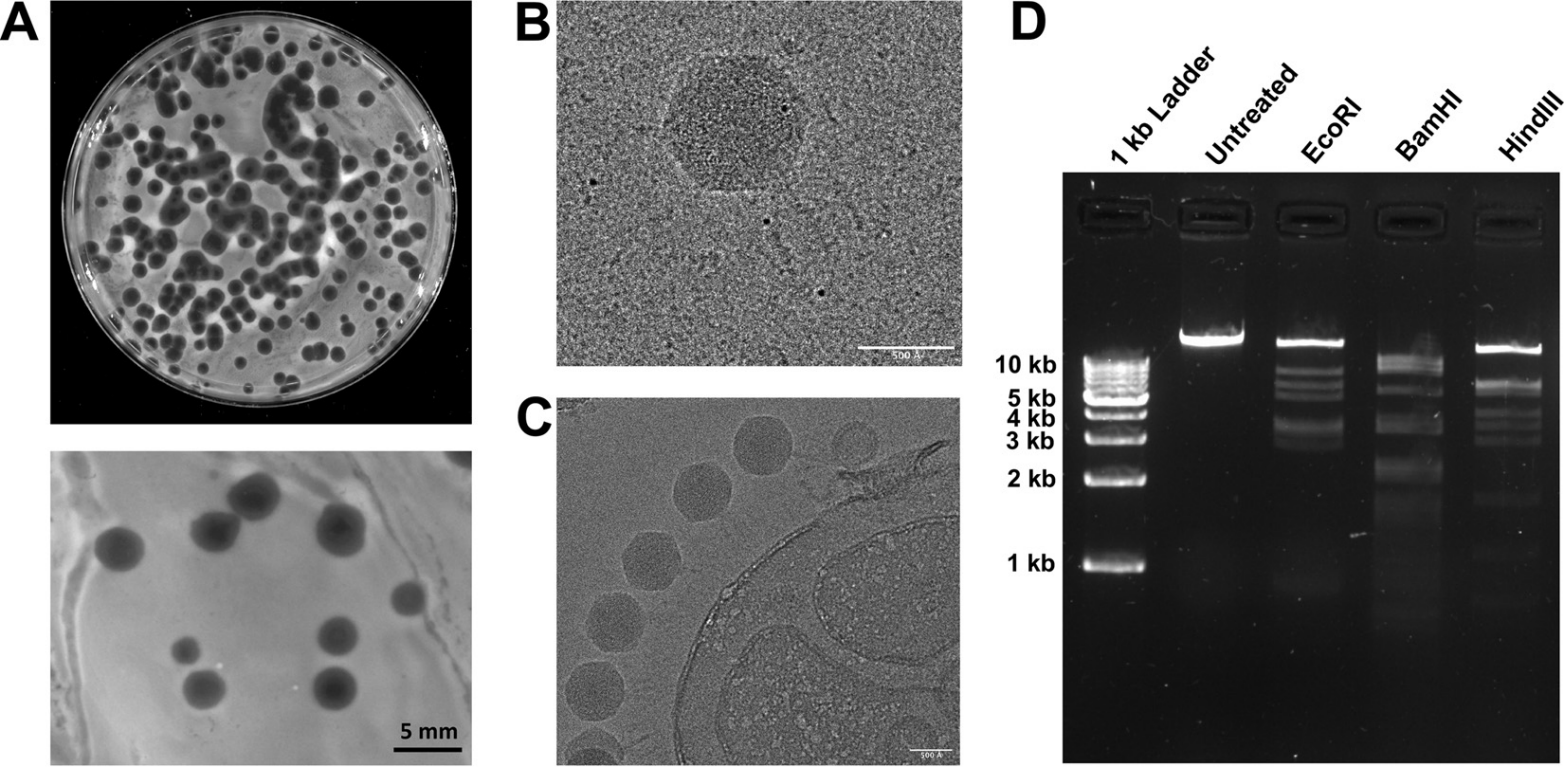
AM.P2, a *Podoviridae* phage isolated from wastewater, produces plaques against PAO1. (A) Isolated phage AM.P2 against host strain PAO1, plated by a double-layer agar method to visualize individual plaques. AM.P2 produces large plaques (5-mm diameter) against PAO1 with clear centers and turbid edges when plated with 0.7% soft agar. (B) Cryo-EM raw image of the AM.P2 phage with the capsid and the tail. (C) Image of several AM.P2 phage particles attached to a bacterial cell-like structure. (D) Restriction digestion of AM.P2 nucleic acid with EcoRI, BamHI, and HindIII.

P. aeruginosa is a ubiquitous organism found in the environment and the human gut. We sampled sewage, known to carry a large load of P. aeruginosa, as a source for anti-P. aeruginosa bacteriophages. Phage AM.P2 was isolated from a wastewater sample in Kollam, Kerala, India, and produced large plaques against the frequently studied model P. aeruginosa strain PAO1 of approximately 5-mm diameter with clear centers and turbid edges (Fig. 1A). Larger plaques often correlate to smaller phage head sizes, as smaller phage particles can diffuse through agar more easily (15).

Initial phage nomenclature and classification is dependent on phage virion morphology and characterization of phage genetic material. Double-stranded DNA-tailed phages are considered to be of the order *Caudovirales*, which consists of three families, *Siphoviridae*, *Myoviridae*, and *Podoviridae*, differentiated based on virion morphology. Cryo-electron micrographs (cryo-EM) of AM.P2 virions revealed phage head and tail dimensions of 716 Å and 235 Å, respectively (Fig. 1B and C). The short, noncontractile tail, as seen in electron micrographs of AM.P2, is characteristic of podoviruses. Digestion of AM.P2 genetic material with restriction enzymes EcoRI, BamHI, and HindIII further confirmed that it is a double-stranded DNA virus (Fig. 1D). Taken together, these findings categorize AM.P2 as a member of the *Podoviridae* family.

Phages are typically considered highly specific in nature due to the unique interactions of individual phage tail fibers with particular host receptors. However, there are certain phages capable of infecting more than a single bacterial host, termed broad-host-range phages. AM.P2 was enriched from sewage using PAO1 as a host but did not exhibit plaque-forming activity against the other clinically relevant bacterial pathogens Klebsiella pneumoniae, Acinetobacter baumannii, Escherichia coli, and Staphylococcus aureus (Table 1).

**TABLE 1:** AM.P2 is a *Pseudomonas*-specific phage

Bacteria	Source	Strain	Lysis
Gram negative			
Pseudomonas aeruginosa	Laboratory strain	PAO1	Yes
Escherichia coli	Laboratory strain	MTCC 40	No
	Sewage (MDR)	ST155	No
Klebsiella pneumoniae	Lab strain	ATCC 33495	No
	Clinical isolate (MDR)	K2	No
Acinetobacter baumannii	Lab strain	ATCC 15308	No
Gram positive			
Staphylococcus aureus	Clinical isolate	MSSA1	No
	Clinical isolate	MSSA2	No
	Clinical isolate (MDR)	MRSA1	No
	Clinical isolate (MDR)	MRSA2	No

### AM.P2 efficiently inhibits PAO1 growth at low MOI.

**FIG 2:**
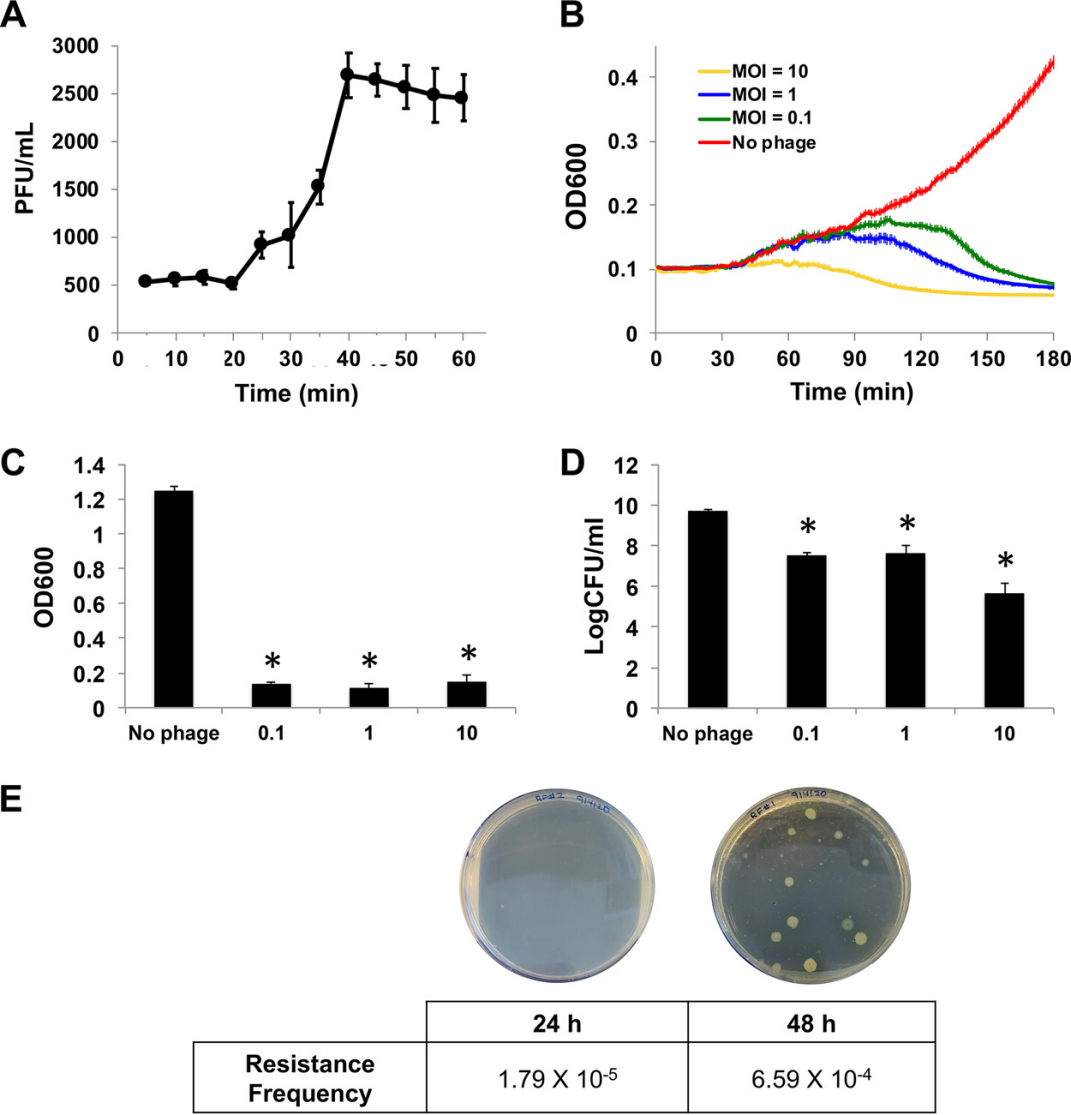
AM.P2 inhibits PAO1 growth and maintains decreased cell viability at MOIs as low as 0.1. (A) AM.P2 one-step growth curve suggests a latent phase of 20 min for PAO1 infectivity. (B) Bacterial growth curve of PAO1 treated with AM.P2 at MOIs of 10, 1, and 0.1. The OD_600_ was measured every minute for 3 h. (C and D) The effect of AM.P2 treatment at the same MOIs after 24 h on OD_600_ (C) and on number of CFU/ml (D). PAO1 without phage was considered the control. Average values of triplicate readings from three independent experiments are plotted with SEM. Significance was determined using *t* test (*, *P*  < 0.05). (E) Resistance frequency of PAO1 to AM.P2 at 24 and 48 h.

To understand the kinetics of the phage infection cycle, a one-step growth curve of AM.P2 was performed with its host, PAO1. Results suggest that AM.P2 rapidly replicates within the bacterium with a latent phase of approximately 20 min (Fig. 2A), consistent with the pattern usually seen with lytic bacteriophages. To better understand the kinetics of AM.P2 activity toward PAO1, bacterial growth inhibition assays were performed at various multiplicities of infection (MOIs) of phage (10, 1, and 0.1). Over a 3 h time frame, AM.P2 rapidly inhibited bacterial growth in a dose-dependent manner, with lower MOIs taking longer to achieve growth inhibition than higher MOIs (Fig. 2B). The AM.P2 MOI of 10 almost immediately inhibited PAO1 growth, whereas declining MOIs of 1 and 0.1 took 80 min and 100 min, respectively, to achieve growth inhibition. At all three MOIs, a significant decrease in cell viability was maintained over an extended 24-h period, ultimately resulting in a 3- to 4-log reduction in numbers of CFU (Fig. 2C and D). The AM.P2 phage resistance frequency was determined as 1.79 × 10^−5^ after 24 h and 6.59 × 10^−4^ after 48 h of phage treatment of PAO1 (Fig. 2E).

### Supplementation of subinhibitory ciprofloxacin concentrations enhances AM.P2 efficacy to reduce PAO1 viability after 24 h.

**FIG 3:**
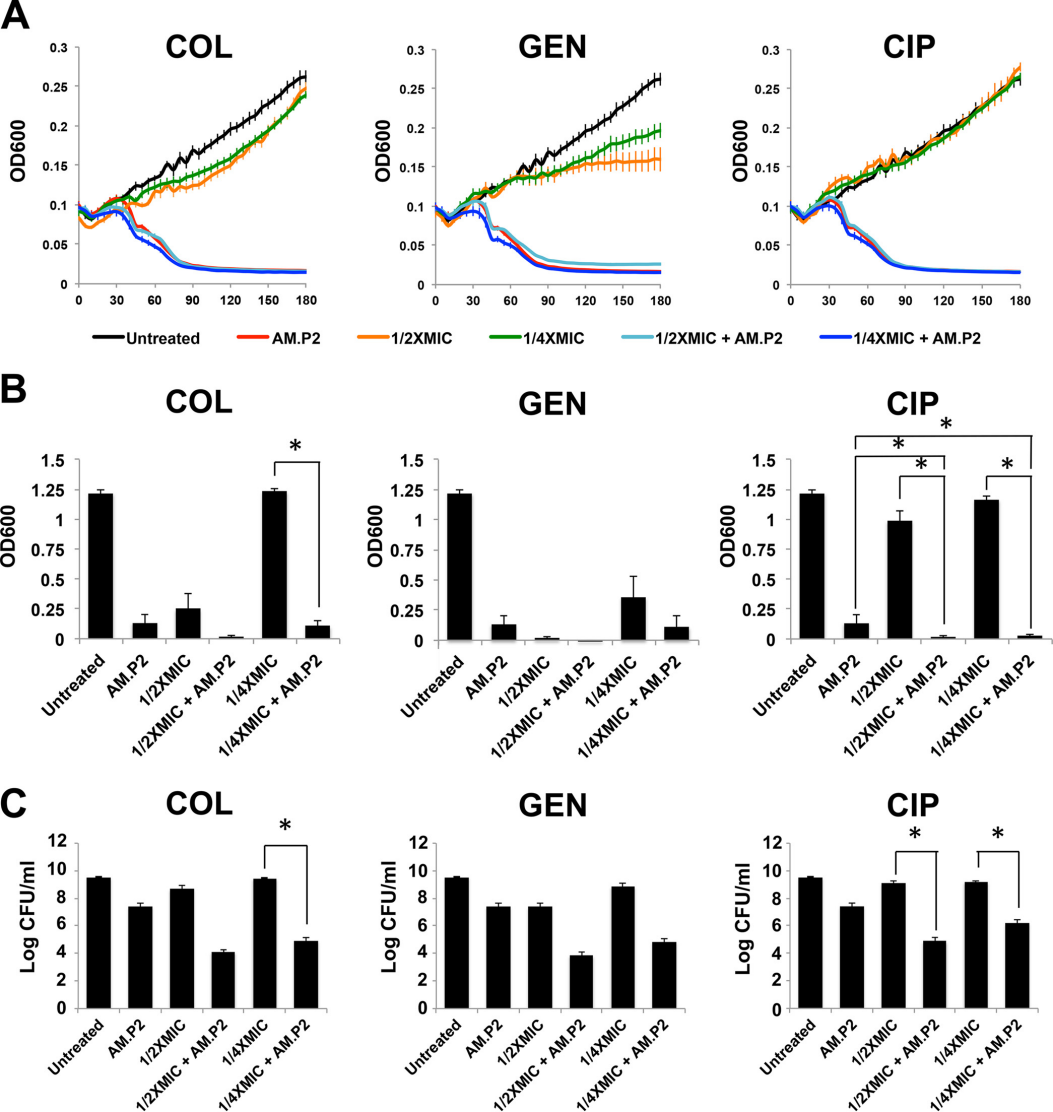
Effect of combinatorial AM.P2 and subinhibitory antibiotic concentrations on PAO1. The effect of PAO1 treated with AM.P2, antibiotics (colistin [COL], gentamicin [GEN], and ciprofloxacin [CIP]) at 1/2×MIC and 1/4×MIC, and combinations of AM.P2 and sub-MIC levels of each antibiotic was assessed by measuring the OD_600_ over the first 3 h of treatment (A), OD_600_ after 24 h of treatment (B), and number of CFU/ml after 24 h of treatment (C). Average values of triplicate readings from three independent experiments are plotted with SEM. Significance was determined using a one-way ANOVA (*, *P*  < 0.05).

Phage resistance has always been a point of concern for the ultimate success of phage applications. One method to overcome the increase in phage-resistant mutants is the combination of antibiotics with phages to lower the number of viable bacteria after treatment. We studied the efficacy of AM.P2 in combination with antibiotics. PAO1 was treated with AM.P2 (MOI of 10) and three classes of antibiotics (a polymyxin, aminoglycoside, and fluoroquinolone), each with different mechanisms of action (interference with bacterial cell membrane integrity, protein synthesis, and DNA replication, respectively). MIC_90_ values for growth inhibition were obtained for colistin (2 μg/ml), gentamicin (4 μg/ml), and ciprofloxacin (0.25 μg/ml) (Table 2), and for combinatorial studies, subinhibitory concentrations of 1/2×MIC and 1/4×MIC for each antibiotic were tested. The rapid decrease in optical density at 600 nm (OD_600_) characteristic of AM.P2 treatment was mirrored in the antibiotic-phage combination treatments, suggesting that supplementation with an antibiotic did not impact phage efficacy in the initial cycles of phage replication and infection (Fig. 3A). However, after 24 h the combinatorial treatment did result in lowered OD_600_ values and bacterial viability compared to single-component treatments (Fig. 3B and C), reaching statistical significance in the case of ciprofloxacin treatment. This suggests that even subinhibitory concentrations of a fluoroquinolone antibiotic synergizes with AM.P2 against P. aeruginosa.

**TABLE 2:** MICs of colistin, gentamicin, and ciprofloxacin against PAO1

Antibiotic	MIC_90_ (μg/ml)	1/2×MIC_90_ (μg/ml)	1/4×MIC_90_ (μg/ml)
Colistin	2	1	0.5
Gentamicin	4	2	1
Ciprofloxacin	0.25	0.125	0.06

### AM.P2 is a N4-like luzseptimavirus closely related to KPP21 and LUZ7.

**FIG 4:**
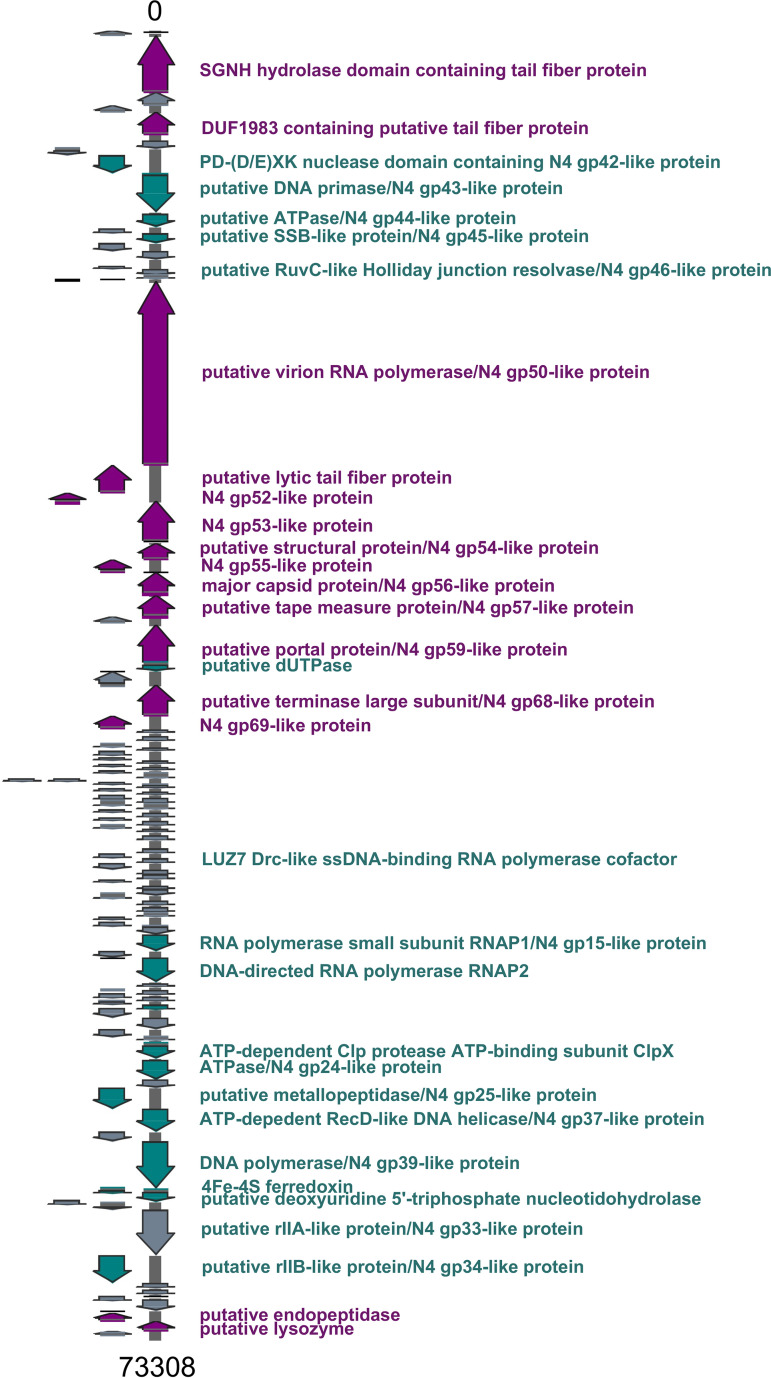
AM.P2 genome map. AM.P2 has a genome of 73,308 bp with a GC content of 53.54%, 110 coding DNA sequences, and 10 rho-independent terminators. Gene calling was done using Glimmer3, MetaGeneAnnotator, and Sixpack, and functional annotation was done using BLASTP with the NCBI nonredundant database and the Swiss-Prot and TrEMBL databases. Terminators were identified using TransTermHP.

Sequence analysis revealed that AM.P2 has a genome of 73,308 kb with a GC content of 53.54% (Fig. 4). In contrast to the general trend of phages with GC content similar to that of their natural host (16), the GC content of AM.P2 differs significantly from that of PAO1 (66.2%). Nucleotide comparison using BLASTN of the full AM.P2 genome assembly delivered two strong hits, KPP21 and LUZ7, among whole bacteriophage genomes. KPP21 and LUZ7 are both N4-like P. aeruginosa bacteriophages. The AM.P2 genome has 110 coding sequences, of which 78 are for hypothetical proteins and 10 for rho-independent terminators (see Table S1 in the supplemental material). Many of the genes with functional annotation have a high percent identity to previously annotated N4-like proteins, supporting the designation of AM.P2 as an N4-like phage. The absence of an integrase-like protein suggests that AM.P2 is a strictly lytic phage. Additionally, the AM.P2 genome is organized such that the early and middle genes are located on the sense strand, and late genes, responsible for phage packaging, are on the antisense strand.

10.1128/mSphere.01215-20.1 TABLE S1 Features of the vB_Pae_AM.P2 genome. Download Table S1, XLSX file, 0.07 MB. Copyright © 2021 Menon et al. 2021 Menon et al. This content is distributed under the terms of the Creative Commons Attribution 4.0 International license.

**(i) Early genes.** The AM.P2 genome encodes many N4-like proteins involved in phage DNA replication, including a PD-(D/E)XK nuclease domain-containing protein/N4 gp42-like protein, putative DNA primase/N4 gp43-like protein, putative ATPase/N4 gp44-like protein, putative single-strand binding protein (SSB)-like protein/N4 gp45-like protein, and putative RuvC-like Holliday junction resolvase/N4 gp46-like protein.

**(ii) Middle genes.** Genes required for transcription, including the RNA polymerase small subunit RNAP1/N4 gp15-like protein and DNA-directed RNA polymerase RNAP2, are found further downstream on the sense strand of the AM.P2 genome. Sequences encoding a putative rIIA-like protein/N4 gp33-like protein and putative rIIB-like protein/N4 gp34-like protein are also present. A homolog of the LUZ7 Drc protein is present in the genome. Drc was recently elucidated as an SSB transcription factor that interacts with RNAP2, playing a role in the transition of early- to mid-phase gene transcription (17).

**(iii) Late genes.** Genes involved in phage assembly are mostly found on the antisense strand. These include the major capsid protein, portal protein, and tail fiber proteins. One specific tail fiber protein contains an SGNH hydrolase domain, which suggests lipase activity. The AM.P2 genome encodes the characteristic N4-like large virion-associated RNA polymerase (vRNAP), which in N4 is coinjected with phage DNA during infection and responsible for the transcription of early genes (18).

**FIG 5:**
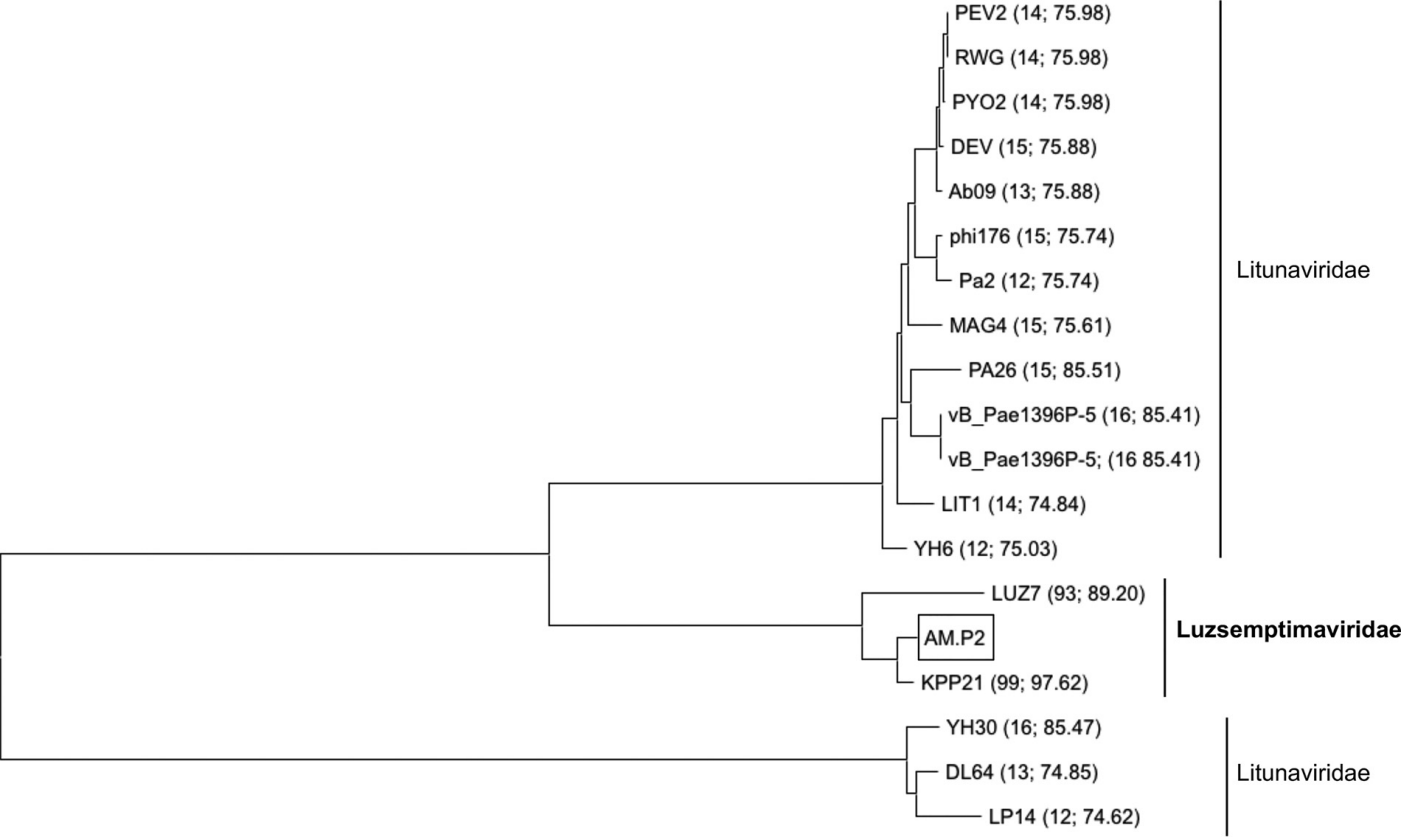
Comparative genomic analysis of AM.P2 and other *Pseudomonas* luszeptimaviruses and litunaviruses. A BLASTN search of the AM.P2 genome against the NCBI representative RefSeq database resulted in 18 phages with high percent identity to the AM.P2 genome. Whole genomes were aligned using ClustalW, and a phylogenetic tree was constructed using the neighbor-joining method in MEGA X. Each phage is labeled with the percent query cover followed by the percent identity to AM.P2 in parentheses.

To assess the characterization of AM.P2 with respect to previously documented phages, a comparative study of the AM.P2 genome was done. Phylogenetic analysis suggests that the phage is closely related to LUZ7 and KPP21 (19, 20), two phages isolated from geographically distant regions (Belgium and Japan, respectively) but that nevertheless share high levels of homology (Fig. 5). According to the NCBI Taxonomy Browser, LUZ7 and KPP21 are the only members of the genus *Luzseptimaviridae*, a group of N4-like P. aeruginosa phages. Two bacterial or archaeal viruses are considered the same species when they share more than 95% nucleotide sequence similarity (21). BLASTN search of AM.P2 with KPP21 and LUZ7 genomes indicates that AM.P2 shares 97% identity with KPP21 and 89% identity with LUZ7, suggesting AM.P2 is a strain of a KPP21-like virus but a distinct species from LUZ7. Members of the *Luzseptimaviridae* differ from the closely related litunaviruses, such as LIT1 and PEV2, as they harbor a 29-small-gene cluster of hypothetical proteins with unknown function, explaining the divergence from a common ancestor of the two genera.

### AM.P2 inhibits growth of MDR P. aeruginosa clinical strains.

**FIG 6:**
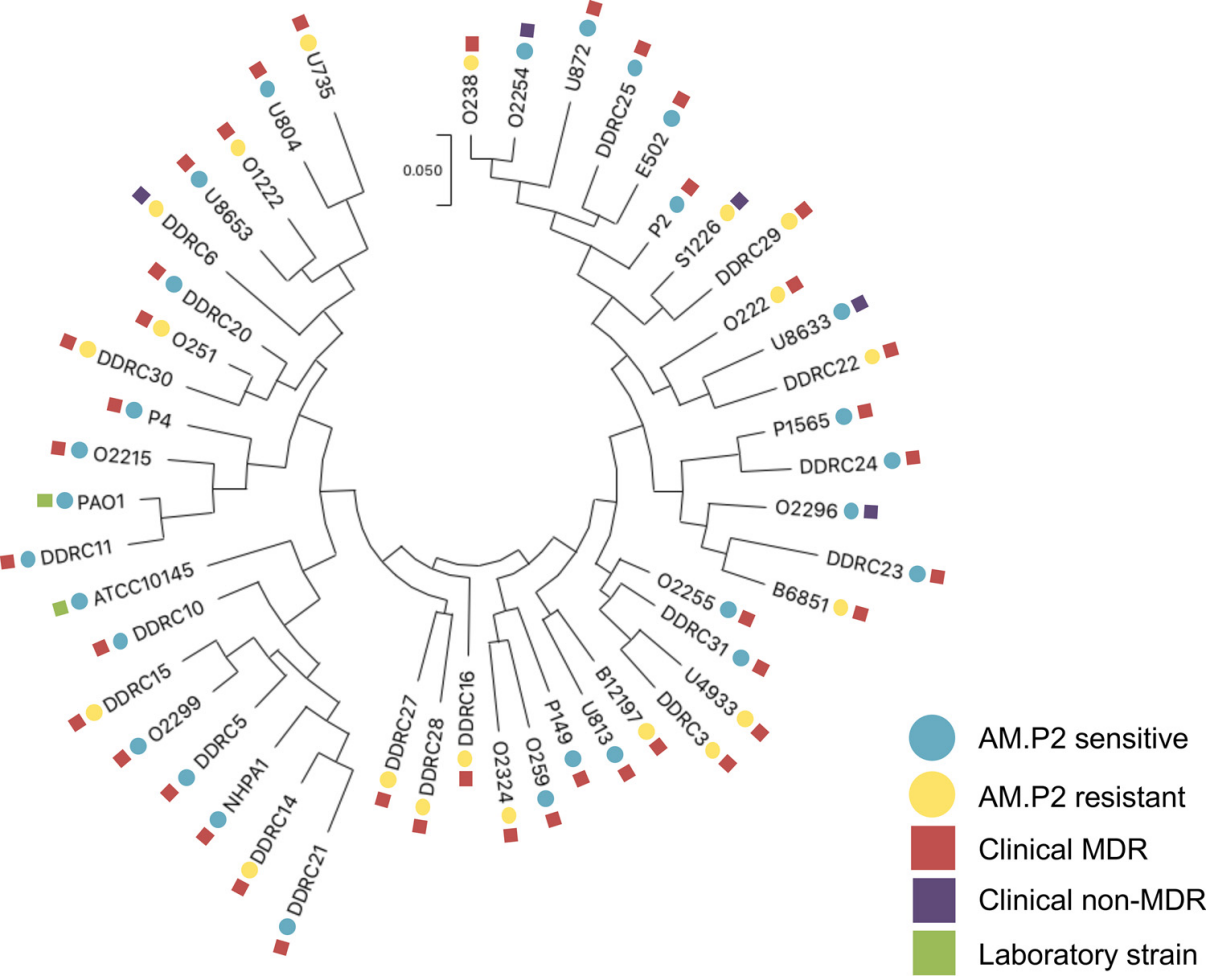
Phylogenetic analysis of AM.P2-sensitive and -resistant clinical strains of Pseudomonas aeruginosa. Spot tests of AM.P2 against 41 clinical MDR and seven antibiotic-susceptible P. aeruginosa strains were done as three independent experiments. Circles indicate strain AM.P2 sensitivity (blue) and resistance (yellow), while squares indicate strain type, such as clinical MDR (red), clinical non-MDR (purple), and laboratory (green) strain.

Although P. aeruginosa is a ubiquitous organism found in the environment, it also can be a dangerous opportunistic pathogen that is often highly antibiotic resistant. To explore the potential application of AM.P2 against such disease-associated strains, tropism was assessed against 48 different strains of P. aeruginosa from different sources. Forty-five strains were isolated from various patient samples from multiple tertiary care hospitals in Kerala, India, out of which 40 were considered MDR and five were non-MDR (Table S2). An additional tested MDR strain (P4) was originally isolated in the New York metropolitan area (22), while the remaining two strains were standard laboratory and genetic reference strains, PAO1 and ATCC 10145. Phylogenetic analysis of these strains showed significant diversity, and AM.P2 produced plaques on 58.3% (28/48) of all the strains tested, including 56.1% (23/41) of the MDR clinical strains (Fig. 6).

10.1128/mSphere.01215-20.2 TABLE S2 Antibiotic susceptibility profiles for clinical isolates of P. aeruginosa. Download Table S2, XLSX file, 0.1 MB. Copyright © 2021 Menon et al. 2021 Menon et al. This content is distributed under the terms of the Creative Commons Attribution 4.0 International license.

**FIG 7:**
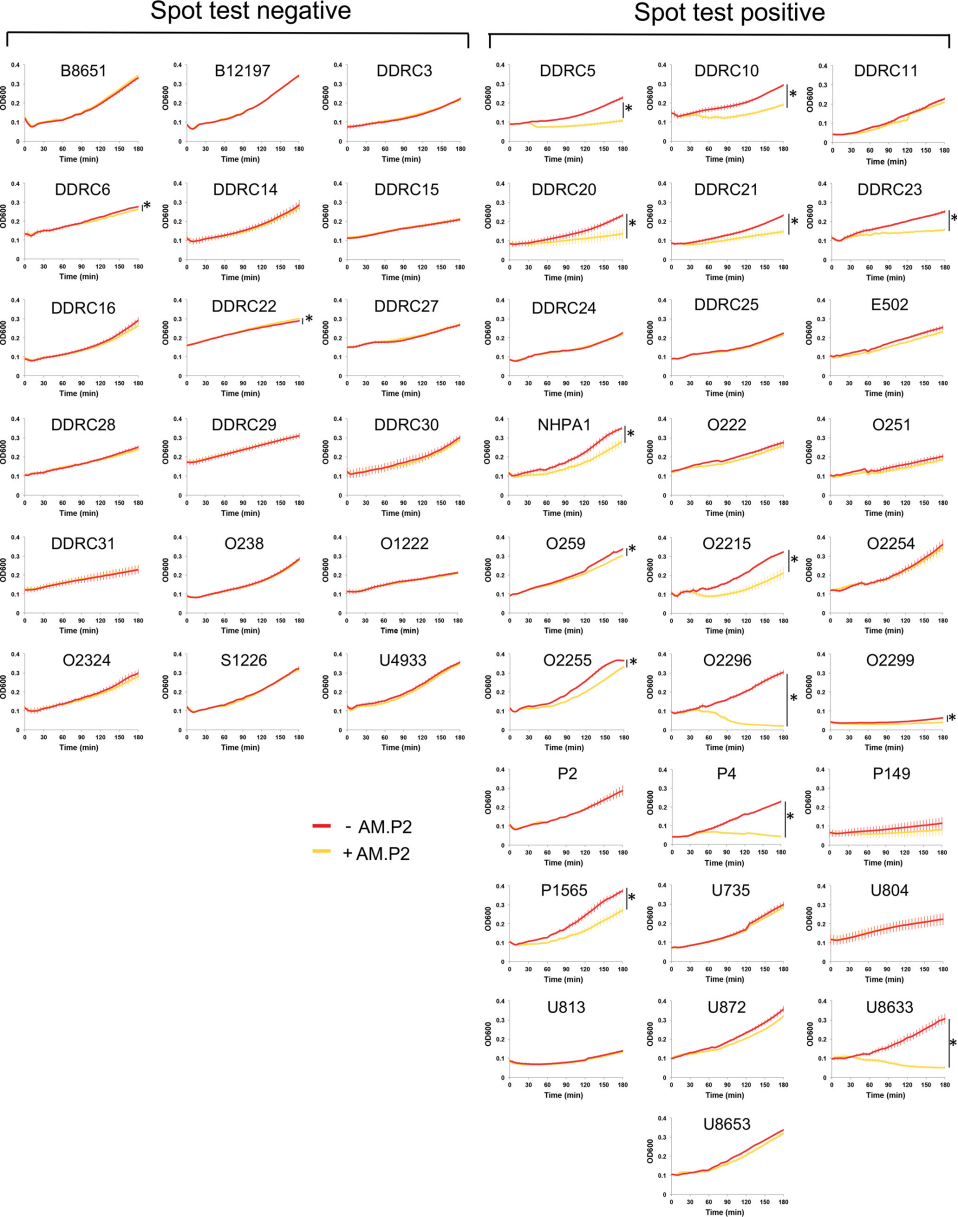
AM.P2 inhibits the growth of MDR P. aeruginosa clinical strains. Bacterial growth curves of the 46 clinical P. aeruginosa strains treated with AM.P2 at an MOI of 10 over a period of 3 h. Average values of triplicate readings from two independent experiments are plotted with SEM. A *t* test was used to determine significant differences in the levels of OD_600_ at *t* = 3 h (*, *P*  < 0.05).

Spot tests were performed using high titers of concentrated phage. To determine if phage efficacy was maintained at lower MOI, treatment of the entire panel of clinical P. aeruginosa strains with AM.P2 at an MOI of 10 was monitored over 3 h (Fig. 7). Notably, half of the strains that were reported as sensitive to AM.P2 in the spot test for plaque formation showed no susceptibility to AM.P2 in the growth inhibition assay. When defining phage sensitivity as a significant decrease in OD_600_ after 3 h, only 26.82% of the MDR clinical isolates could be defined as AM.P2 sensitive. The apparent discrepancy between the results of the two assays indicates that high bacteriophage titers are required for efficient lysis of some clinical isolates.

## DISCUSSION

Although there is a lack of consistent statistics on antimicrobial resistance (AMR) data from the Indian subcontinent, reports describing the incidence of resistance in community-acquired infections show undoubtedly higher rates than those in developed nations. The Indian AMR scenario is marked by a significantly high prevalence of MDR pathogens, thereby providing a potential model environment for studying the rapid rise and spread of MDR bacterial populations and uncovering possible solutions. Researchers and clinicians alike are exploring alternative and adjuvant therapies for the treatment of MDR P. aeruginosa infections. Expanding the phage repertoire is beneficial for the scientific and medical communities, and AM.P2, a Pseudomonas aeruginosa N4-like phage isolated from sewage wastewater, is an intriguing candidate phage to add to the arsenal of known phages to be used for varied applications.

Bacteriophage therapy has shown increasing promise and has been successful in treating multiple cases of MDR Gram-negative infections that showed little to no hope of recovery through traditional antibiotic treatment modalities (23–26). vB_Pae_AM.P2, a bacteriophage isolated from sewage in Kerala, India, can to inhibit the growth of a range of Pseudomonas aeruginosa strains. We report here that AM.P2 efficiently lyses laboratory strain PAO1, with a 3- to 4-log decrease in number of CFU compared to that of untreated PAO1. Unfortunately, bacteria are continually evolving and adapting, and just as antibiotic resistance arises, phage resistance is also inevitable. AM.P2, like any other lytic bacteriophage, cannot be viewed as a silver bullet for the complete eradication of P. aeruginosa, as bacteriophage-insensitive mutants do arise after an extended period of phage-bacterium interaction. Proposed methods to overcome phage resistance include the implementation of polyphage cocktails or phage-antibiotic combinatorial treatments, both of which have resulted in successful outcomes in *in vitro* and *in vivo* tests (27). The current study underlines the potential importance of combinatorial treatment with antibiotics, highlighted by the finding that treatment of P. aeruginosa with ciprofloxacin at a subinhibitory concentration along with AM.P2 results in greater bactericidal activity than treatment with either antibiotic or phage alone. When used in combination with AM.P2 in the management of MDR infections, perhaps dose-sparing regimens of the antibiotics could be selected.

AM.P2 is a P. aeruginosa-specific phage that infects numerous South Indian clinical P. aeruginosa isolates, many of them resistant to multiple classes of antibiotics. Spot tests are a simple yet generally accepted primary assessment of a strain’s phage susceptibility. However, through a comparison of the results from the AM.P2 spot tests with growth inhibition assays to determine host tropism, it appears that these assays give somewhat different results, with the latter reporting fewer strains as phage sensitive. While a spot test can answer the yes-or-no question regarding whether bacteria can be lysed by a phage, sensitive assays using lower titers may be more informative in assessing the ability of a phage to sensitize bacteria at more relevant titers for various applications. Despite the apparent discrepancy in the cumulative correlation between these different phage sensitivity assays, it is clear that AM.P2 possesses the ability to infect and lyse MDR P. aeruginosa clinical isolates.

This study reports the phenotypic and genotypic characterization of AM.P2, a P. aeruginosa-specific, lytic bacteriophage. Future studies could focus on understanding the molecular mechanism of infection and translating our findings to practical applications. Common phage receptors include flagella, pili, or lipopolysaccharide, all of which contribute to bacterial virulence. A connection between virulence, phage sensitivity, and synergizing antibiotics may provide a basis for the optimal selection of candidates for appropriate phage applications. Previous studies suggested that laboratory-derived phage resistance is mostly accounted for by mutations in host receptor genes and is not due to CRISPR-mediated resistance that is prevalent in environmental settings (28). Future studies to elucidate the AM.P2 phage receptor could help identify the mutations that may be correlated with phage resistance and if these could pose a survival trade-off for the virulence of the pathogen. Additionally, further *in vivo* experimental models can be explored to truly assess the efficacy of this phage in therapeutic applications.

## MATERIALS AND METHODS

### Ethics approval.

This study was approved by the Institutional Ethics Committee, Amrita Institute of Medical Sciences (IEC-AIMS-2019-SBT-097). All experiments with DNA isolated from clinical strains were also approved by the TIGS Institutional Biosafety Committee (IBSC) review board (2019). Clinical strains were obtained from patients in tertiary care hospitals as part of the routine care done for patients without any additional procedures.

### Bacterial strains.

Clinical strains were collected from multiple tertiary health care centers over a period of approximately 2 years, spanning from September 2017 to November 2019. These strains were provided on blood agar and subsequently subcultured on cetrimide agar. Single colonies were grown in Luria-Bertani (LB) broth and stored as glycerol stocks. All strains were routinely grown in LB broth aerobically (shaking at 200 rpm) at 37°C.

### Antibiotic susceptibility testing.

All clinical isolates were subjected to antibiotic susceptibility testing against gentamicin, amikacin, tobramycin, ciprofloxacin, levofloxacin, ceftazidime, piperacillin, ticarcillin, aztreonam, imipenem, meropenem, and colistin by the Kirby Bauer disc diffusion method using Pseudomonas aeruginosa strain ATCC 27853 as a control. Briefly, bacteria were grown to an OD_600_ of 0.1 in 3 ml LB, and 300 μl of each culture was swabbed on the surface of Mueller-Hinton agar plates. Antibiotic discs (HiMedia) were placed on the swabbed culture incubated for 16 to 18 h at 37°C, following which the zones of inhibition were measured, and each strain was determined as resistant/intermediate/sensitive to each antibiotic tested according to the chart provided by the manufacturer. Strains that were resistant to three or more classes of antibiotics were considered MDR.

### AM.P2 isolation and purification.

AM.P2 was isolated from a sewage wastewater sample near Kollam, Kerala, using a previously described protocol (29). Briefly, 80 ml of wastewater sample was added to 9 ml of 10× enrichment broth (10× nutrient broth plus 0.5 M K_2_HPO_4_) and 3 ml of overnight culture of PAO1 and enriched aerobically for phages at 37°C for 16 to 24 h. The enriched sample was centrifuged twice at 6,500 × *g* for 10 min, and the supernatant was treated with 1:100 chloroform and/or filtered through a 0.22-μm filter. The resulting lysate was diluted and plated by the double-agar overlay method to yield single plaques, as previously described (30). To purify for a single phage population, isolated plaques were selected and replated five times.

### Preparation of AM.P2 high-titer stocks.

For high-titer phage stocks, AM.P2 was enriched in a plate by molten streaking on soft agar with PAO1 (31). After 16 to 24 h of incubation at 37°C, 5 ml of SM buffer was added on the surface of the agar for 30 min. The SM buffer, now containing phage particles, was aspirated into 1.5-ml microcentrifuge tubes and centrifuged at 10,000 × *g* for 5 min to remove bacterial debris, and the supernatant was treated with 1:100 chloroform for 10 min. For further concentration, AM.P2 was precipitated by adding 300 μl of 5× polyethylene glycol (PEG) 8000-NaCl (20% PEG-8000 and 2.5 M NaCl) to 1,200 μl of phage sample. After overnight incubation at 4°C, sample was centrifuged at 20,000 × *g* for 20 min, and the resultant phage pellet was resuspended in 200 μl of SM buffer.

### Cryoelectron microscopy.

Virus particles were applied to a glow-discharged R1.2/1.3 holey carbon grid (Quantifoil Micro Tools GmbH, Germany), blotted for 3 s with a blot force of 0, and plunge-frozen in liquid nitrogen-precooled ethane using a Vitrobot Mark-IV (Thermo Fisher Scientific, USA) at 100% humidity. Two virus samples, arising from different purification batches, were imaged on a Titan Krios 300 KV microscope equipped with a Falcon 3 direct electron detector. The first sample was imaged at ×47,000 magnification, corresponding to a pixel size of 1.78 Å. The second sample was imaged at ×59,000 magnification, which corresponds to a pixel size of 1.38 Å. Final images were processed using ImageJ (32).

### One-step growth curve.

Phage growth kinetics and latent period were determined by one-step growth curve experiments, performed as previously described, with a few modifications (33, 34). Briefly, 100 μl of AM.P2 (5 × 10^6^ PFU/ml) was added to 9.9 ml of PAO1 grown in LB broth to an OD_600_ of 0.4. After adsorption for 5 min, 100 μl of AM.P2 plus PAO1 was added to 9.9 ml of fresh LB (A) and further diluted by transferring 1 ml to 9 ml of LB (B). At 5-min intervals over a span of 1 h, 100-μl aliquots were taken from both A and B, mixed well with 100 μl PAO1 culture and 5 ml LB soft agar (0.7%), and plated by the double-agar overlay method. Mean numbers of PFU/ml from three independent experiments were plotted along with standard errors of the means (SEM).

### MIC determination.

MICs were determined using the microdilution method for the antipseudomonal antibiotics colistin, gentamicin, and ciprofloxacin as described previously, with some modifications (35). PAO1 was grown to an OD_600_ of 0.2 in LB and then diluted 1:100. Twenty microliters of the diluted culture and 20 μl of 10× the desired antibiotic concentration were added to each well, and the total volume was made up to 200 μl in LB. After 24 h of static incubation at 37°C, absorbance at OD_600_ was measured using an Enspire Alpha multimode plate reader (PerkinElmer). MIC_90_ was determined as the minimum concentration of antibiotic that would inhibit bacterial growth by ≥90% compared to the untreated bacterial control.

### Bacterial growth inhibition assays.

Bacterial growth inhibition assays were done as previously described, with slight modifications (36). For the 3 h kinetics assay, 180 μl of bacterial culture grown in LB to an OD_600_ of 0.2 was mixed with 20 μl of 10^9^, 10^8^, and 10^7^ PFU/ml AM.P2 to maintain MOIs of 10, 1, and 0.1, respectively, in a 96-well plate. For phage-antibiotic combinatorial studies, 160 μl of bacterial culture was mixed with 20 μl of 10^9^ PFU/ml AM.P2 and 20 μl of 10× the desired concentrations of colistin, gentamicin, or ciprofloxacin. No phage controls were treated with SM buffer, and no antibiotic controls were treated with LB. Plates were incubated at 37°C for 3 h, and bacterial growth was measured by checking absorbance at OD_600_ every minute for PAO1 and every 5 min for all other strains/phage-antibiotic combinations using a plate reader. For 24-h incubations, plates were set up in the same manner, except the starting culture (OD_600_ of 0.2) was diluted 1:100 and mixed with 20 μl of 10^7^, 10^6^, and 10^5^ PFU/ml AM.P2; 96-well plates were incubated at 37°C without shaking for 24 h. Final absorbance at the OD_600_ was measured using a plate reader. Mean triplicate values from three independent experiments were plotted along with SEM.

### Bactericidal activity assays.

Bactericidal activity assays were done as previously described, with slight modifications (35, 37). Briefly, 96-well plates were set up as mentioned previously for the 24-h bacterial growth inhibition assay. After 24 h, serial 10-fold dilutions were performed for each well in sterile phosphate-buffered saline (PBS). Twenty microliters of each serial dilution was spot plated on LB plates and incubated overnight at 37°C and plotted along with SEM.

### Phage resistance frequency determination.

AM.P2 resistance frequency was determined per the previously described protocol (38). PAO1 was grown to an OD_600_ of 0.2 and diluted 1:100 in LB. Eighteen microliters of diluted culture was combined with 20 μl of 10^7^ PFU/ml AM.P2 (MOI of 10) in 14-ml culture tubes. Tubes were incubated with shaking at 37°C for 10 min to allow for phage adsorption. Four milliliters of LB soft agar (0.7% agar) was added to the tubes, mixed well, poured on the surface of an LB plate, and allowed to solidify before incubating at 37°C. The number of bacteriophage-insensitive mutants (BIMs) was enumerated after 24 and 48 h. Resistance frequency was calculated at these time points by dividing the number of BIMs by the number of CFU in the starting culture.

### AM.P2 genomic DNA isolation.

Genomic DNA from 200 μl of high-titer AM.P2 stock was isolated using phenol-chloroform. Briefly, PEG-precipitated phage was centrifuged at 20,000 × *g* for 20 min, and the pellet was resuspended in 200 μl 0.05 M Tris-Cl, pH 8. The sample was incubated with 2 μl of DNase (2,000 U; Ambion) and 2 μl of RNase (10 mg/ml; Thermo Scientific) for 1 h at 37°C. An equal volume of buffered phenol (10 mM Tris, 1 mM EDTA) was added and tubes were mixed well, followed by collection of the aqueous layer after centrifugation at 10,000 × *g* for 2 min. This was followed by the addition of 200 μl of chloroform, centrifugation at 10,000 × *g* for 2 min, and collection of the aqueous layer. Twenty microliters of 3 M sodium acetate (pH 4.8) and 400 μl of 100% molecular-grade ethanol were added, and the precipitated DNA was pelleted at 10,000 × *g* for 10 min. After a 70% ethanol wash, the pellet was allowed to dry and then resuspended in 30 μl of nuclease-free water. DNA was quantified using a NanoDrop spectrophotometer (Thermo Fisher).

### Whole-genome sequencing.

For Illumina sequencing, phage DNA was prepared with a Nextera XT DNA library preparation kit according to the manufacturer’s instructions. Sequencing was done at the National Centre for Biological Science (NCBS), Bangalore, India, on an Illumina HiSeq 2500 platform with 100-bp paired-end reads and ∼1,000× coverage. The library for Nanopore sequencing was prepared using a one-dimensional (1D) ligation sequencing kit (SQK-LSK109) from Oxford Nanopore Technologies (ONT). Library preparation was a two-step process that included end-prep and adapter ligation. End-prep was performed to convert the overhangs into blunt ends and to adenylate the 3′ ends of the DNA, followed by cleanup. Adapter ligation, specific for the R9.4.1 flow cell chemistry run, was performed by adding the adapter mix and the T4 ligation kit from the 1D ligation sequencing kit to the dA-tailed DNA, followed by another cleanup. The DNA library was mixed with sequencing buffer and loading beads immediately before loading. The sample was loaded gently, in a dropwise fashion, into the SpotON sample port in the flow cell prepared for sequencing and sequenced using the MinION device. The sequencing was initiated and run for an hour with high-accuracy base calling using the MinKNOW software. Guppy was used for base calling to obtain the final sequence output in the Fastq format.

### Genome assembly and annotation.

Nanopore reads were assembled using Canu, which performs three operations, correction, trimming, and assembly, in a sequential manner to generate the final assembled contigs. The Nanopore assembly functioned as a scaffold for the Illumina assembly. Both the Nanopore assembly and the Illumina short reads were uploaded into the Galaxy web platform, and a final assembly was done using SPAdes (version 3.12.0) in the Galaxy Center for Phage Technology (CPT) server (https://cpt.tamu.edu/galaxy-pub) (39, 40). Multiple contigs were created, but only one had appropriate coverage, which, after PCR confirmation, was considered the true AM.P2 phage genome. Annotation was done in the Galaxy CPT interface using the PAP structural workflow 10.v8 and PAP functional workflow v8.16. Briefly, possible genes were identified using Glimmer3 (41–43), MetaGeneAnnotator (44), and Sixpack (45). Terminators were called using TransTermHP, and, per the CPT Galaxy training for phage annotation (https://cpt.tamu.edu/training-material/topics/phage-annotation-pipeline/tutorials/structural-annotation-workflow/tutorial.html), false positives that scored less than 90, had less than a 5-bp stem without mismatch, and had fewer than four Ts following the stem-loop were filtered out manually (46). ARAGORN was used to detect tRNAs (47). Functional annotation was done with BLASTP with the NCBI nonredundant database and Swiss-Prot and TrEMBL databases (48–50). The genome map was constructed using SnapGene v. February 2020.

### Phylogenetic analysis of AM.P2.

The AM.P2 genome was aligned to the representative RefSeq genome database to search for similar genomes using BLASTN. The top 18 hits that had >70% identity and >10% query cover were selected for further analysis. Multiple-sequence alignment of whole phage genomes was done using ClustalW, and phylogenetic trees were constructed using the neighbor-joining method in MEGA X (45, 51).

### Phylogenetic analysis of P. aeruginosa clinical strains.

Bacterial DNA was isolated using the DNeasy UltraClean microbial kit per the manufacturer’s instructions. Regions of 16S rRNA were amplified with Q5 high-fidelity DNA polymerase (NEB) and universal primers (27F, AGAGTTTGATCCTGGCTCAG; 1492R, TACCTTGTTACGACTT) (52). The PCR protocol was initial denaturation at 95°C for 3 min, 35 cycles of denaturation at 95°C for 30 s and annealing at 52°C for 1 min, and extension at 72°C for 1 min. A final extension of 72°C for 2 min was followed by a hold at 4°C. The resulting amplicons were purified using the QIAquick PCR purification kit, and Sanger sequencing was done at the National Centre for Biological Science (NCBS), Bangalore, India. The forward and reverse reads for each strain were aligned using BLASTN, and the final 16S rRNA sequences from all strains were aligned using ClustalW. The resulting alignment was imported into MEGA X, and the evolutionary history was inferred using the maximum likelihood method and Tamura-Nei model. The phylogenetic tree was constructed by applying neighbor-joining and BioNJ algorithms to a matrix of pairwise distances estimated using the maximum composite likelihood (MCL) approach and then selecting the topology with superior log likelihood value.

### Tropism assay.

Spot tests were done by first swabbing an overnight culture of each strain on an LB plate and allowing it to dry. Two microliters of concentrated AM.P2 stock (>10^10^ PFU/ml) was spotted and allowed to dry. Plates were incubated overnight at 37°C and observed for lysis.

### Statistics.

The significance of the effects of AM.P2 on P. aeruginosa was determined using paired *t* tests. The effect of combined AM.P2 and antibiotic treatment compared to single components was deemed significant by means of a one-way analysis of variance (ANOVA) followed by a *post hoc* Tukey’s test. Statistical significance was defined as a *P* value of  <0.05 (*).

### Data availability.

All genome sequencing data have been deposited under BioProject no. PRJNA629366 and in the NCBI BioProject database under BioSample no. SAMN14794289. The Illumina and Nanopore reads were deposited as Sequence Read Archive (SRA) accession no. SRR11665926 and SRR11772201, respectively, and the AM.P2 complete genome as GenBank accession no. MT416090.
